# A Novel Qualitative and Quantitative Biofilm Assay Based on 3D Soft Tissue

**DOI:** 10.1155/2014/768136

**Published:** 2014-02-18

**Authors:** Bodil Hakonen, Linnea K. Lönnberg, Eva Larkö, Kristina Blom

**Affiliations:** ^1^Medibiome AB, 431 53 Mölndal, Sweden; ^2^Department of Medical Microbiology and Immunology, Sahlgrenska Academy, University of Gothenburg, 413 45 Göteborg, Sweden

## Abstract

The lack of predictable *in vitro* methods to analyze antimicrobial activity could play a role in the development of resistance to antibiotics. Current used methods analyze planktonic cells but for the method to be clinically relevant, biofilm in *in vivo* like conditions ought to be studied. Hence, our group has developed a qualitative and quantitative method with *in vivo* like 3D tissue for prediction of antimicrobial activity in reality. Devices (wound dressings) were applied on top of *Pseudomonas aeruginosa* inoculated Muller-Hinton (MH) agar or 3D synthetic soft tissues (SST) and incubated for 24 hours. The antibacterial activity was then analyzed visually and by viable counts. On MH agar two out of three silver containing devices showed zone of inhibitions (ZOI) and on SST, ZOI were detected for all three. Corroborating results were found upon evaluating the bacterial load in SST and shown to be silver concentration dependent. In conclusion, a novel method was developed combining visual rapid screening and quantitative evaluation of the antimicrobial activity in both tissue and devices. It uses tissue allowing biofilm formation thus mimicking reality closely. These conditions are essential in order to predict antimicrobial activity of medical devices in the task to prevent device related infections.

## 1. Introduction

There is a plethora of different *in vitro* methods that evaluates antimicrobial activities. One common method is minimum inhibitory concentration (MIC). This method analyzes the activity of planktonic cells but not cells in biofilm. Biofilms are considered to be the natural way of existing for bacterial cells and were reported in 2003 by National Institute of Health (NIH) to cause over 80% of all infections [1]. It has also been realized that cells in biofilms are 50–1000 times less susceptible to antibiotics [2, 3]. Despite the growing evidence that infections are due to aggregates of bacteria, that is, biofilm, antibiotics are evaluated in planktonic cell assays risking false positive effect *in vitro* and in the worst case no clinical effect *in vivo* contributing to erroneous therapeutic value of antibiotics and drive of increasing antibiotic resistance [4].

Biofilms are problematic also in device related infections (DRI). DRI are reported to constitute up to 60% of health care associated infections where devices such as catheters, endotracheal tubes, and implants are the most implicated. To prevent infections, medical device manufacturers put resources to develop surface modifications with antimicrobial properties. For instance, St. Jude Medical (St. Paul, MN) developed silver coated heart valves in their efforts to decrease the number of fatal infections [1]. However, it was found that the incidence was higher among the coated devices than the uncoated ones due to that biofilm formation was promoted on silver coated rather than uncoated devices [5]. This observation was missed since the manufacturer had evaluated the antimicrobial activity with a method using planktonic cells and not biofilm cells. Hence, care must be taken in how these tests are designed. Preferably, if clinical predictability is sought, antimicrobial properties should be tested with methods close to reality.

Several *in vitro* biofilm methods are based on solid substratum such as 1.5% agar [6] or 30% poloxamer [7]. However, these substrata are made of polysaccharides and are not close to the clinical setting where the main component is collagen, a major protein in soft tissues existing in, for example, skin.

Yet another important aspect is that many devices are in contact with soft tissue. Therefore, when designing a clinical relevant method for DRI, the interactions between the device, bacteria, and surrounding tissue are essential for full comprehension. Our group has developed a novel method based on 3-dimensional (3D) soft tissue allowing biofilm formation [8] where the antimicrobial activity can as a first step be visualized and then if required quantified. The initial visual step is advantageous for quick and easy screening and the quantification step enables definite measure on the antimicrobial and/or antibiofilm activity. This method analyzes the antimicrobial activity on microorganisms both on the device and in surrounding tissue.

## 2. Materials and Methods

### 2.1. Bacterial Strain and Culture Condition

*Pseudomonas aeruginosa* (PAO1), ATCC 15692, was incubated overnight at 35 ± 2°C in tryptic soy broth (TSB) (Oxoid, Basingstoke, England) and diluted in simulated wound fluid (SWF; Substratlab, Göteborg, Sweden) containing 1 : 1 fetal calf serum and 0.1% peptone water to 10^6^ cells/mL, to obtain the start inoculum.

### 2.2. Preparation of Muller Hinton Agar

12-well polystyrene plates (NUNC, Roskilde, Denmark) were cast with Muller-Hinton (MH) agar (Oxoid). Briefly, 7.6 g MH agar powder was dissolved in 200 mL water, autoclaved, and poured into each well of the 12-well plates and allowed to solidify.

### 2.3. Preparation of Collagen Based 3D Synthetic Soft Tissue

SST was prepared according to a previously published protocol [9]. 12-well polystyrene plates (NUNC) were cast with SST made of polymerized rat-tail collagen type I (BD Biosciences, San Jose, CA) containing 11% serum proteins. Briefly, 12 mL collagen matrix at 2 mg/mL was prepared under cooled conditions; 2.62 mL SWF was mixed with 1.2 mL of 10x phosphate buffered saline (PBS) and 0.18 mL 1 M NaOH to which 8.0 mL collagen solution (3 mg/mL in 0.02 N acetic acid) was added. The collagen matrix was added to each well of 12-well plates and to achieve complete polymerization the plate was placed at 37°C for 1 hour.

### 2.4. Preparation of Test Specimens

In this study, test specimens were wound dressings with silver and their counterparts without silver: Aquacel Ag/Aquacel (ConvaTec, Skillma, NJ), Mepilex Ag/Mepilex (Mölnlycke Health Care, Göteborg, Sweden), and Allevyn Ag/Allevyn (Smith & Nephew, Hull, UK). These dressings are henceforth nominated as hydrofiber (HF), silver containing HF (HF-Ag), soft silicon foam (SSF), silver containing SSF (SSF-Ag), foam (F), and silver containing F (F-Ag) in respective order as listed above. All dressings were punched to 5 mm in diameter.

### 2.5. Biofilm Formation on MH Agar and 3D Synthetic Soft Tissue (SST)

30 *μ*L bacterial suspensions were inoculated to cover the whole surface of MH or SST and incubated at 35°C for 24 hours to allow biofilm formation.

### 2.6. Qualitative Assessment of Antibacterial Activity

Test specimens were applied on top of inoculated MH agar or SST and incubated at 35°C for 24 hours. Presence of zones of no growth was documented by photography.

### 2.7. Quantification of the Bacterial Load

SST was digested by mixing the punched gel exposed to the test specimen with 60 *μ*L collagenase (Biochrome AG, Berlin, Germany) at 500 *μ*g/mL in PBS followed by incubation at 35°C for 1.5 hours. After the enzymatic digestion, the samples were neutralized and serial diluted in neutralization buffer (Dey Engley; Fluka from Sigma-Aldrich) followed by plate counts.

### 2.8. Statistical Analysis

All values presented in graphs are expressed as mean ± SEM (standard error of the mean). Statistical significance was determined between silver containing and placebo for each type of dressing using Welch's *t*-test. *P* values ≤ 0.05 were considered statistically significant.

## 3. Results

### 3.1. Qualitative Assessment

**Figure 1:**
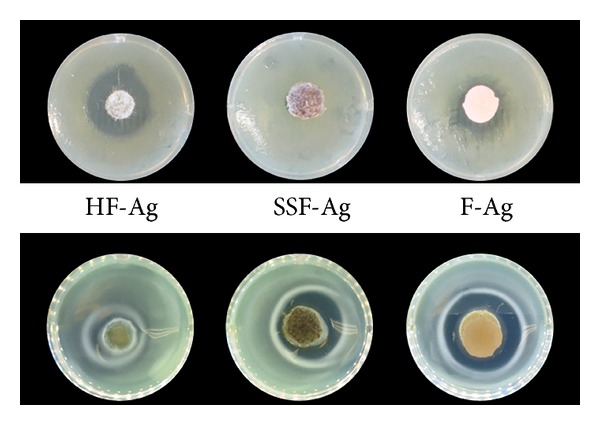
Test specimens containing different silver compounds were applied on Muller-Hinton (MH) agar (top row) as well as on 3D synthetic soft tissue (SST) (bottom row).

PAO1 was spread on MH agar plates and on collagen based 3D SST, respectively. Test specimens were applied in the center and after 24-hour incubation the antibacterial activity was evaluated. When using MH agar two out of three test specimens showed zones and when using SST, zones were detected for all specimens (Figure 1).

### 3.2. Quantification of Bacterial Load in 3D Synthetic Soft Tissue (SST) and Dressings

**Figure 2:**
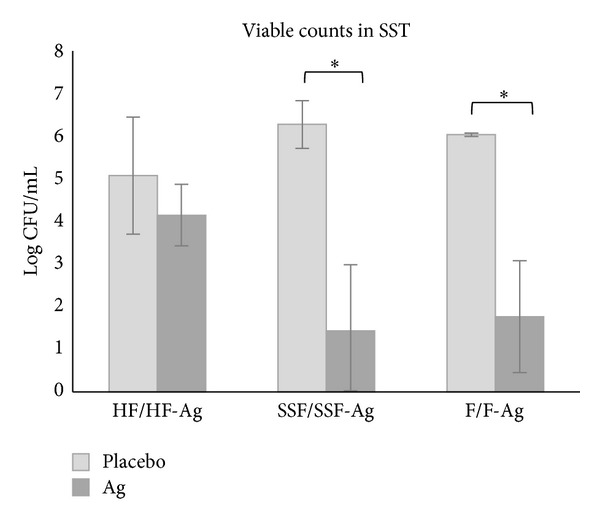
Graph showing bacterial load in 3D synthetic soft tissue (SST) after 24-hour exposure to wound dressings without (placebo) and with silver (Ag). Error bars show standard error of the mean (SEM) with *n* = 3 for the placebo samples (except *n* = 2 for F) and *n* = 6 for the silver containing dressings. *Significantly different from corresponding placebo at *P* < 0.05.

The bacterial activity was also analyzed quantitatively. SST was completely digested and viable counts could be obtained. It was found that the bacterial load in the SST was reduced for all silver containing products compared with their counterparts of their respective placebos and statistically lower for SSF-Ag and F-Ag treated tissue (Table 1, Figure 2).

**Figure 3:**
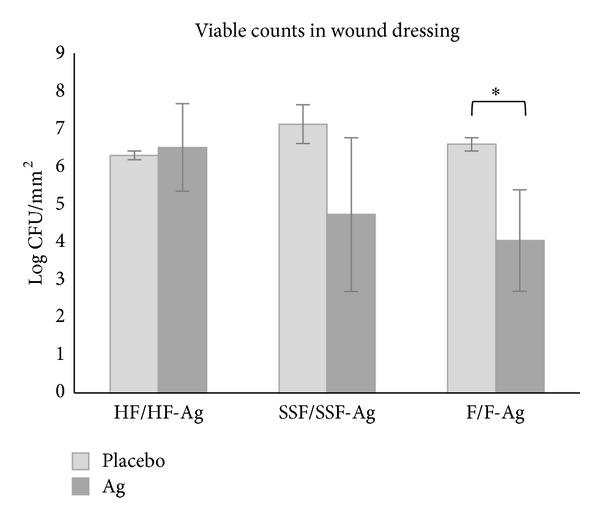
Graph showing bacterial load in wound dressings without (placebo) and with silver (Ag) after 24-hour incubation on inoculated SST. Error bars show standard error of the mean (SEM) with *n* = 3 for the placebo samples and *n* = 5 (HF-Ag and F-Ag) or *n* = 6 (SSF-Ag) for the silver containing dressings. *Significantly different from corresponding placebo at  *P* < 0.05.

Bacterial load in wound dressings were analyzed and found to be at about the same level in all placebos and H-Ag, lower in SSF-Ag and F-Ag, and significantly lower for F-Ag (Table 2, Figure 3).

## 4. Discussion

The rise of increased antibiotic resistance may to some extent be due to that current methods give false positive activity caused by that planktonic cells have been tested and not biofilms. This leads to ineffective treatments since biofilms are 50–1000 times less susceptible to antibiotics [2]. In the fight against rising resistance, the need for methods that can better predict reality is urgent, thereby promoting the development of effective antimicrobials. Hence, our group has developed a novel method where antimicrobial activity can be analyzed both qualitatively and quantitatively using synthetic soft tissue (SST) allowing biofilm formation in a similar manner as in human soft tissue [8]. The verification of that a biofilm is formed in the SST was elegantly shown by Werthén et al. [8] where typical markers for biofilm were proven to exist as well as that the biofilm formation was clinically relevant. The SST consists mainly of collagen type I which is a fibrous extracellular matrix protein, a major component in soft tissue. In contrast, agar (used in the established Kirby-Bauer disk diffusion assay [10]) is derived from red algae and is a polysaccharide widely used as a solidifier to grow bacteria and is not a constituent of soft tissue. In this method SST contains serum proteins as well making this substratum even more *in vivo* like providing a clinical relevant environment whereupon products or substances can be applied and their antimicrobial activity screened visually as well as quantified.

The Kirby-Bauer method is widely used for antibiotic susceptibility testing where filter disks soaked with antibiotics are applied on MH agar inoculated with bacteria [10]. If a zone of inhibitions (ZOI) is created the bacteria are sensitive to the drug. The ZOI test, known as the disk diffusion test, has been adapted to suit products such as wound dressings and has then been referred to as corrected zone of inhibitions (CZOI) test [11]. Then, CZOI equals the zone minus the size of the test specimen. In our method, the same principal is employed but on SST for a fast, visual detection of antimicrobial activity. However, in contrast, we advocate strongly that CZOI should not be used with the aim of comparing different products quantitatively. This is explained by that different products often have different substances with different diffusion coefficients and different release systems affecting the size of the zones. Hence, CZOI should in these instances only be used as a qualitative method. Still, this visual method is highly relevant providing rapid screening indicating if there is any antimicrobial activity and if there is a release of the antimicrobial substance from the product. In addition to wound dressings analyzed in present study, a variety of other products and substances can be screened.

To show proof of concept with this novel method, wound dressings containing silver and their counterparts' placebos were evaluated. The choice of wound dressings is owed partly to that there is an interesting ongoing debate about which method is the most relevant and also partly since certain wound dressings made of foam are prewetted in the CZOI method to show antimicrobial activity. Prewetting is in sharp contrast with the intended clinical use of the dressing where it is applied dry to enable absorption of wound fluid. Therefore, we chose to not apply prewetting in our study. Qualitative analyses revealed clear zones for all silver dressings on SST while zones on MH agar were seen only for HF-Ag and F-Ag but not for SSF-Ag. This difference is probably explained by that water molecules are more easily accessible in the SST than in MH agar, which is true also for real soft tissue. This condition seems essential to allow the release of silver ions from SSF-Ag, which would explain why it was not required to be pre-wetted in our method but in the CZOI test using MH agar (data on file). Thus a fair comparison when running CZOI using MH agar is complicated since different wound dressings require different pre-treatments.

The qualitative assessment offers rapid detection of antimicrobial activity which is useful for screening purposes. In addition to the qualitative visual detection, this novel method also offers a quantitative way to estimate the antimicrobial activity resulting in numerical values. The soft tissue made up of collagen was easily broken down with collagenase freeing bacteria within the matrix thus enabling plate counts. Enumeration of bacterial cells in soft tissue and in the applied devices (wound dressings) was done. It was found that the viable counts were reduced in soft tissue when exposed to silver containing devices compared with placebo (Table 1 and Figure 2). The HF-Ag dressing reduced the bacterial load with log 1 while SSF-Ag and F-Ag with log 5 and log 4, respectively. The greater reduction seen for the silver containing foams is most likely owed to their higher silver content; SSF-Ag at 1.2 mg/cm^2^ and F-Ag at 0.9 mg/cm^2^ compared to HF-Ag at 0.09 mg/cm^2^ [12]. Bacterial survival in the wound dressings was also evaluated (Table 2 and Figure 3). It was found that the silver containing foams had >log 2 less bacterial burden compared with their respective counterparts while the bacterial burden was equivalent in both the HF-Ag and its corresponding device without silver. This indicates that the activity on the bacterial cells is dependent on the concentration of the release of silver. These results are logic; the more silver is released the more antimicrobial activity is seen. However, results from the Kirby-Bauer method contradict logic, probably due to that the release of silver is prevented on agar while it is allowed in the SST. This highlights the need to understand that products have different release systems due to their carrier materials. These different properties should be taken into consideration when choosing what test method to use to show antimicrobial activity.

Another highly relevant feature with our novel method is that the bacterial cells can be grown as *in vivo* like biofilms, the predominant way of living for bacteria. In addition, this method uses soft tissue which makes it closer to reality than MH agar. A lawn of bacterial cells or biofilm will grow on the surface of MH agar in 2 dimensions while as aggregates within the soft tissue in 3D. An increasing body of evidence supports that bacterial biofilms exist also in the wound beds of hard-to-heal wounds pushing the demand for wound dressings with antibiofilm properties. Thereby it has been realized that antimicrobials should be tested against biofilms for predicting the activity *in vivo*. To further mimic the reality, the biofilm should be grown in tissue. The use of humans or animals in wound models brings obvious ethical limitations and practical drawbacks such as larger laboratory facilities and technical expertise required. However, established *in vitro* biofilm methods analyze mainly the formation of biofilm on surfaces of, for example, a device [13–15]. This method also analyzes the effect on the bacteria in the tissue and not only bacteria attached on the test specimen. It should be emphasized that the biofilm formed in the tissue is probably or is even more clinically relevant in the cause of infections. This is highlighted in the event of hip prosthesis infections. The infection will not be resolved by just removing the prosthesis but extensive tissue debridement is also required to remove all infected tissue [16]. We here present a method for analyzing biofilm formation and inhibition, in an *in vivo* like model where different products or substances can be evaluated.

Further development of our method could include human cells, for example, fibroblasts. This would enable the analysis of also toxic effects, such as cytotoxicity and foreign body reaction, but would require additional techniques, for example, histology and immunohistochemistry as well as molecular biology techniques to study gene expression and protein expression of inflammatory markers and growth factors. However, inclusion of cells would increase the cost and time and be much more cumbersome to run. In this present study, our intention was to develop a method that should be rapid, simple, and cost efficient to visually screen and quantify the antimicrobial activity of products or substances in a clinical relevant method which allows biofilm formation.

## 5. Conclusion

Central to *in vitro *methods is that they should predict the clinical situation with the aim of avoiding the use of animals and humans. If predictable, these methods offer useful ways to foretell what activity a certain substance or product has on its target. However, many test methods are far from reality and hence expected effects of a new drug or medical device may be omitted when used clinically. This is often the case when it comes to antimicrobial substances and products, potentially leading to inefficient therapies and resistance. The conflict with reality comes to that antimicrobial activity has been tested on bacterial cells as free living organisms instead of complex aggregates, that is, biofilms that are found *in vivo*. Furthermore, tests are generally done using conditions adapted for bacterial growth rather than running the tests under *in vivo* like conditions. This novel method mimics reality closely owing to the 3D synthetic soft tissue (SST) made of collagen and serum proteins creating an environment that supports biofilm formation. By using this *in vivo *like substratum we succeeded in refining the method of Kirby-Bauer, a novel method which allows rapid visual screening, and in addition quantitative assessment of the antimicrobial effects of tissue as well as on products under *in vivo* like conditions.

## Figures and Tables

**Table 1:** Log reduction of *Pseudomonas aeruginosa* (PAO1) in 3D synthetic soft tissue (SST) treated with silver containing test specimens in comparison to their placebos.

Test specimens	Log reduction
HF-Ag	1
SSF-Ag	5*
F-Ag	4*

*Significantly different from corresponding placebo at *P* < 0.05.

**Table 2:** Log reduction of *Pseudomonas aeruginosa* (PAO1) in silver containing dressings in comparison to their placebos.

Test specimens	Log reduction
HF-Ag	NA^†^
SSF-Ag	2.4
F-Ag	2.5*

^†^NA: not Applicable; there was an increase of growth; hence, no reduction could be calculated. *Significantly different from corresponding placebo at *P* < 0.05.
